# Suspected COVID-19 mRNA Vaccine-Induced Postural Orthostatic Tachycardia Syndrome

**DOI:** 10.7759/cureus.34236

**Published:** 2023-01-26

**Authors:** Nicole Maharaj, Steven Swarath, Rajeev Seecheran, Valmiki Seecheran, Avidesh Panday, Naveen Seecheran

**Affiliations:** 1 Medicine, North Central Regional Health Authority, Port of Spain, TTO; 2 Internal Medicine, University of Kansas School of Medicine, Wichita, USA; 3 Cardiology, The University of the West Indies, St. Augustine, TTO

**Keywords:** postural orthostatic tachycardia syndrome, pfizer-biontech, coronavirus disease 2019, covid-19, mrna vaccine, pfizer-biontech covid-19 vaccine, autonomic dysfunction, dysautonomia, postural orthostatic tachycardia syndrome (pots)

## Abstract

We present a case of a 15-year-old South Asian male who developed suspected postural orthostatic tachycardia syndrome (POTS) two weeks after receiving the Pfizer-BioNTech coronavirus disease 2019 (COVID-19) vaccine booster, which was successfully managed with low-dose fludrocortisone and ivabradine. Clinicians should be aware of the Pfizer-BioNTech COVID-19 vaccine being implicated with the onset of POTS.

## Introduction

The coronavirus disease 2019 (COVID-19) pandemic is unprecedented and resulted in greater than six million deaths worldwide [1]. Messenger ribonucleic acid (mRNA) vaccines have emerged as an acquired immunologic therapy to mitigate COVID-19 and its deleterious effects. These vaccines have demonstrated both clinical safety and efficacy against the virus [2].

Autonomic dysfunction has been reported following infection with COVID-19 [3,4]. Additionally, there have been a few case reports of dysautonomia occurring in patients following vaccination, including postural orthostatic tachycardia syndrome (POTS) and a case of inappropriate sinus tachycardia (IST) [5-9].

We present a case of a 15-year-old South Asian male who developed suspected POTS two weeks after receiving the Pfizer-BioNTech COVID-19 vaccine booster, which was successfully managed with low-dose fludrocortisone and ivabradine.

## Case presentation

A 15-year-old South Asian male patient with no significant medical history presented with recurrent presyncope and syncope to the emergency department for the previous week. Syncope would occur after prodromal presyncope with faintness and lightheadedness and would be brief, often lasting a few seconds, without any epileptic features. He reported, on average, several episodes of each daily, with the latter occurring as many as 10 times per day, each lasting a few seconds in duration. He did not report any recent antecedent viral infection with COVID-19 as of the onset of the pandemic, head trauma, medication use, or travel history. The patient also refuted any lifestyle modification with respect to dietary intake and physical activity and routinely performed his usual activities of daily living. Prior to the onset of symptoms, he was physically active and regularly participated in vigorous sporting activities such as football (soccer) at his local high school. Two weeks earlier, he received the Pfizer-BioNTech COVID-19 vaccine booster with the initial two-dose sequence eight months prior. Additionally, he was also vaccinated with the Gardasil® human papillomavirus (HPV) vaccine three months previously. COVID-19 polymerase chain reaction testing was negative upon admission.

**Figure 1:**
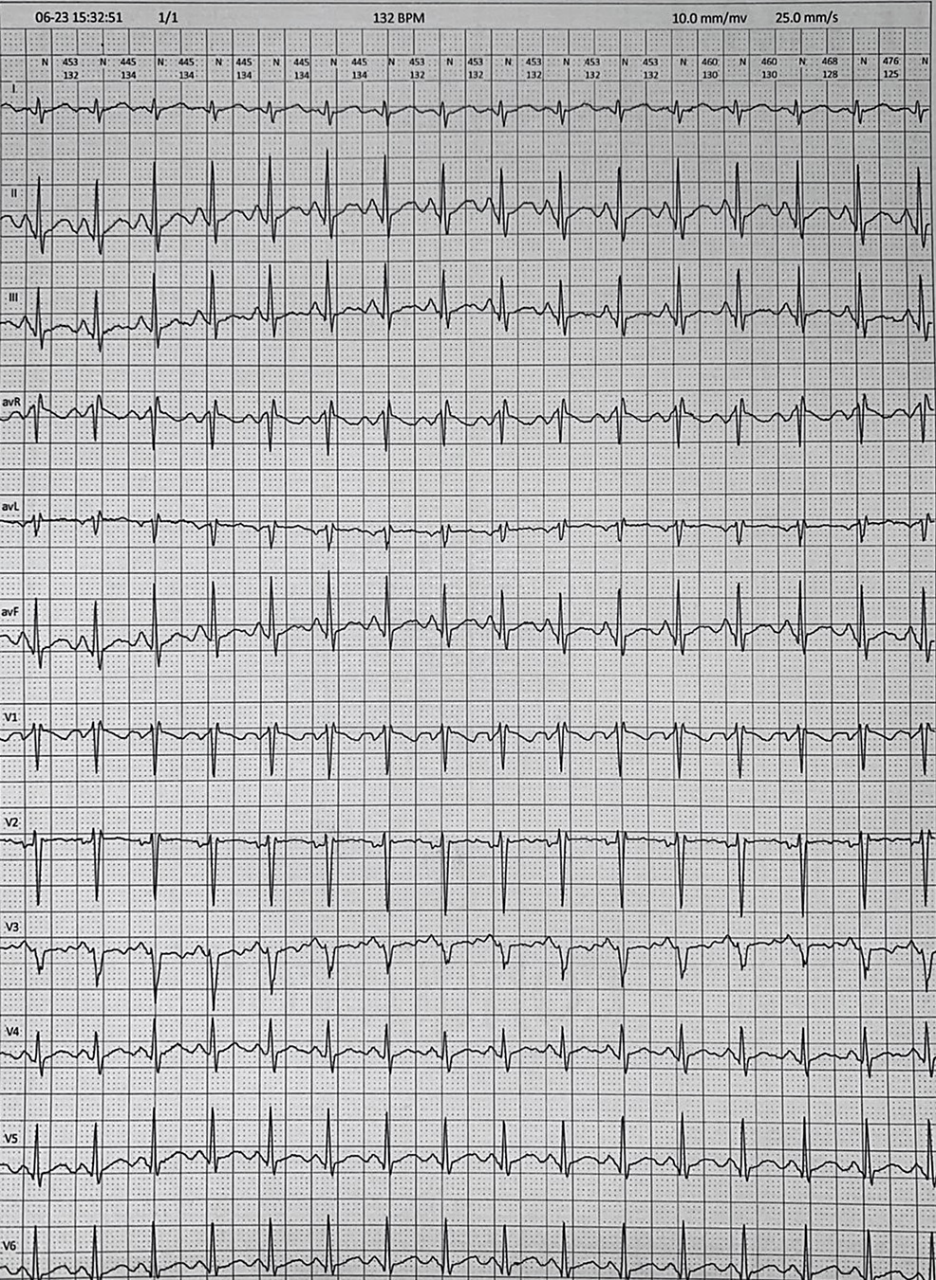
The patient's inpatient telemetry displayed a marked sinus tachycardia, during which he was borderline normotensive but symptomatic with a syncopal episode.

His vital signs on presentation revealed a blood pressure of 105/53 mmHg and a regular heart rate of 107 beats per minute, with pulse oximetry of 100% on room air. He was afebrile with a body mass index of 19.3 kg/m². His orthostatic vital signs were normal. On physical examination, he was alert and oriented without any neurological deficits. His heart sounds were normal, with vesicular breath sounds on auscultation of the lung fields. There was no peripheral edema. Further advanced testing included a two-dimensional transthoracic echocardiogram, computed tomography of the brain, magnetic resonance imaging and angiography of the brain, and 24-hour ambulatory electroencephalogram, all of which were normal. Inpatient telemetry revealed sinus tachycardia during the syncopal episodes (Figure 1). Tilt table testing was not performed due to its unavailability in our setting.

During his ensuing three-day hospitalization, the patient was initiated on fludrocortisone 0.1 mg every eight hours and ivabradine 2.5 mg every 12 hours. He was also advised on lifestyle modification with respect to increasing sodium and fluid intake, avoiding sun exposure to prevent dehydration, and following a moderate-intensity exercise regimen. His constellation of symptoms, including exercise tolerance and fatigue, gradually abated to where he experienced decreasing episodes of syncope on consecutive days, and on the penultimate day and day of discharge, he did not experience any. At one-month post-discharge, the patient's medical regimen was de-escalated to fludrocortisone twice daily as monotherapy, and there were no other serious adverse events.

## Discussion

POTS is a clinical syndrome characterized by the onset of symptoms upon standing, an increase in heart rate of 30 beats per minute in adults (or 40 beats per minute in children and adolescents aged 12-19 years) within 10 minutes of being upright from the supine position, and the absence of orthostatic hypotension. Symptoms commonly include chest pain, dyspnea, lightheadedness, palpitations, exercise intolerance, and fatigue. There is a predilection for women; most cases are apparent from ages 13 to 50 years [10,11]. Emerging research has revealed that POTS is an accentuated response to orthostatic tachycardia. In a healthy patient in an erect position, the effective circulating volume pools due to gravitational force, which elicits a neural reflex for peripheral vasoconstriction, increasing venous return to the heart. In contrast, POTS patients cannot stimulate this response, and the alternative neural compensatory mechanism occurs by increasing the heart rate, alluding to dysautonomia [12,13].

Several conditions, including infection, surgery, and pregnancy, can precipitate or even exacerbate POTS. The chief etiology is usually post-infection, which has been reported with COVID-19. Although post-COVID-19 autonomic pathophysiology is complex and has not been fully elucidated, autoimmunity is thought to be a critical aspect of POTS. Several reports have demonstrated that COVID-19 antibodies cross-react with autonomic nervous system components, such as ganglia, nerve fibers, and G-protein-coupled receptors, which can trigger dysfunction [3,11]. The mRNA vaccine induces native host immunity to produce the viral spike protein antigen, subsequently eliciting an adaptive humoral immune response [2]. Sequelae of these cross-reacting antibodies possibly include interfering with α1-adrenergic receptors leading to a maladaptive vasoconstrictor response and increased sympathetic activation [5]. Additionally, the role of angiotensin-converting enzyme 2 (ACE2) receptor dysfunction has been implicated in the etiology of POTS, as COVID-19 attenuates ACE2 receptor activity and consequently upregulates sympathetic activation [14].

POTS has been described following both mRNA-based Moderna COVID-19 and Pfizer-BioNTech COVID-19 vaccines [5-8]. Interestingly, the cases associated with the latter usually occur one to three weeks post-inoculation, a similar time frame to our patient. Most of the other patients did not have any significant comorbidities apart from major depressive disorder, hypothyroidism, and B12 deficiency, which were not seen in our patient [5,7]. There seemed to be a predilection for the female gender and younger and middle-aged patients, as most cases included the 17-53 age group. POTS has also been reported post-HPV vaccine, which occurs on average ~90 days (but even exceeding a year) after receiving the vaccine in patients aged 12-32 years [15-17]. Our patient received the HPV booster three months prior to symptom onset, which could be considered a confounder. A recent epidemiologic population-based Danish study failed to find any causal association between HPV vaccination and POTS, and the American Autonomic Society stated that current data do not implicate HPV vaccination as an etiology of dysautonomia [16,17].

POTS management is multifaceted to suppress cardiovascular dysfunction and mitigate symptoms [18]. Eight to 10 glasses of water are generally recommended [11]. Gradual physical training also improves symptoms [18]. If the patients display refractory symptoms, off-label pharmacotherapeutics include beta-blockers, midodrine, and fludrocortisone; however, no medication has been approved by the US Food and Drug Administration. Sinus node blocker ivabradine can inhibit the chronotropic response without exerting any hypotensive effects, and this was demonstrated in approximately 60% of patients who experienced symptom improvement [10,19,20].

To our knowledge, this particular case is unusual, as our patient appears to be the youngest (15 years old) of those reported in association with a COVID-19 mRNA-based vaccine, male, and the only person of South Asian ethnicity to display this syndrome. In contrast, the other patients' age ranged from 17 to 53 years, and they were females and of predominantly Caucasian and East Asian ancestry. Our patient displayed all of the salient diagnostic criteria for POTS, including orthostatic symptomatology of presyncope and syncope, the tachycardic response of greater than 40 beats per minute, as evidenced by ambulatory telemetry indicating a heart rate of 132 beats per minute, and without overt hypotension, as measured by several modalities as per the American Heart Association guidelines [21]. As aforementioned, the patient did not report any changes in dietary habits or physical activity, both of which could be implicated as potential distractors. Our patient also received the HPV booster three months prior to symptom onset, which could be considered a confounder. He was successfully managed with both pharmacological and non-pharmacological measures with appropriate endocrinology follow-up to assess for any adrenal gland suppression and crises.

## Conclusions

We presented the case of a 15-year-old South Asian male who developed suspected POTS two weeks after receiving the Pfizer-BioNTech COVID-19 vaccine booster, which was successfully managed with low-dose fludrocortisone and ivabradine. Clinicians should be aware of the Pfizer-BioNTech COVID-19 vaccine being implicated with the onset of POTS.
